# Supplementary material for: Uncertainty estimation for trust attribution to speed-of-sound reconstruction with variational networks
Source: Int J Comput Assist Radiol Surg. 2025 Jun 10;20(7):1541–9. doi: 10.1007/s11548-025-03402-4 (PMC12226603; doi:10.1007/s11548-025-03402-4)
Supplement: Supplementary file 1 — (pdf 557 KB) [file 11548_2025_3402_MOESM1_ESM.pdf]

Uncertainty Estimation for Trust Attribution to Speed-of-Sound Reconstruction with Variational Networks
— *Supplementary Material* —

Sonia Laguna, Lin Zhang, Can Deniz Bezek, Monika Farkas, Dieter Schweizer, Rahel A. Kubik-Huch, Orcun Goksel

1 Loss Definition and Exponential Weighting

To minimize gradients vanishing in the last layers (loops) during training, a weighted loss with exponential decrease of τ was used [1]:

$$\mathcal{L}_{\mathrm{T}} = \sum_{k=1}^{K} \exp^{-\tau(K-k)} \|x_k - x^*\|_1 + \lambda_r \sum_{k=1}^{K} \sum_{j=1}^{N_{\mathrm{j}}^{\phi_{\mathrm{k}}}} \sqrt{\left(y_{j-1}^{\phi_k} - 2y_j^{\phi_k} + y_{j+1}^{\phi_k}\right)^2 + \varepsilon}. \quad (1)$$

The second part of the loss corresponds to a smoothing of the transformation functions ϕ_k, where $N_{\mathrm{j}}^{\phi_{\mathrm{k}}}$ is the number of knots for transformation function parametrization with the value $y_j^{\phi_k}$. Note that ε is added for stability. The regularization weight λ_r is used to balance smoothing and reconstruction losses. We refer the reader to [2] for the detailed derivation of the data gradient and explanation of the network structure.

2 Data Preprocessing and Implementation Details

Figure S1 provides a comparative overview of the steps followed for the data generation and preprocessing processes. The simulated data is obtained based on given ground truth SoS maps via two approaches as described in Section 3.1 in the main paper. Overall, the final displacement d is the input to the VN and is acquired in the different ways shown below in both simulated and *in vivo* data. Table S1 includes a comprehensive list of the parameters used in the proposed methods.

SoS and pixel-wise uncertainty estimation processes described in *Section 2.2* of the main manuscript are formalized here in Algorithm S1. Computations of the per-frame metrics from pixel-wise uncertainty estimates, *i.e.* the minimum uncertainty in the

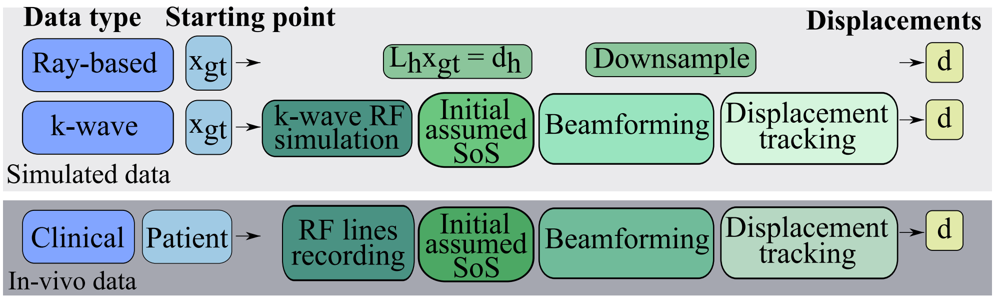

Fig. S1 The displacement generation pipeline for the three datasets in this project. The first two simulation pipelines are for ray-based and k-wave data, where the ground truth is the starting point. The last row is for the *in vivo* data, where the RF recordings are the starting point as the ground truth is unknown.

Table S1 Parameters dimensions and hyperparameter values for the VN model and BVI & MCD implementations.

Parameter	Description	Dimension
w_k	Spatial weights	$57 \times 77 \times 32$
r_k	Regularizers as convolutional filters	$[8, 8, 32]$
m_k	Dropout masks in MCD for convolutional filters	$[8, 8, 32]$
$\tilde{\mu}_{r_k}$	Mean values of convolutional filters	32
ϕ_k	Regularization term activation	35 knots per layer
ψ_k	Data term activation	35 knots per layer
**Hyperparameter**	**Description**	**Value**
K	Number of layers	20
N_k	Number of filters	32
τ	Exponential weighting	5
λ_r	Activation function smoothing	10^5
p	Dropout probability in MCD	0.25
o	Kernel size in BVI	8
α	Prior for weight learning in BVI	0.1
β	Kl regularization weight in BVI	10

inclusion and the relative uncertainty of the inclusion normalized by the background, are described in Algorithm S2. Frame selection based on these per-frame uncertainty surrogates is detailed in Algorithm S3. A code snippet of the efficient algebraic reformulation introduced in *Equation (5)* in the main manuscript for the KL term of the BVI loss is included in Tensorflow in Figure S2.

```
BVI_cost = (alpha_BVI * tf.linalg.trace(tf.matmul(st_dev_mat,
tf.transpose(st_dev_mat)))- 2 * tf.trace(tf.log(st_dev_mat))
```

Fig. S2 Python code snippet using Tensorflow for cost-efficient computation of the KL term in BVI, as in *Equation (5)* in the main manuscript.

Algorithm S1 Uncertainty Estimation for SoS Reconstruction

Require: Trained Variational Network (VN); input displacement data d; number of samples K.
Ensure: Reconstructed SoS image (c) and uncertainty (Uncertainty).

procedure UNCERTAINTY ESTIMATION(Method)
    if Method == MCD **then**
        Set dropout probability p during inference (same as at training).
        for $i = 1$ to K **do**
            Apply dropout mask to the VN.
            Compute i'th SoS reconstruction map $c_i = \mathrm{VN}(d)$.
        end for
    else if Method == BVI **then**
        VN with filters as Gaussian distributions with mean μ and covariance Σ.
        for $i = 1$ to K **do**
            Sample weights $\theta_i \sim \mathcal{N}(\mu, \Sigma)$.
            Compute i'th SoS reconstruction map $c_i = \mathrm{VN}(d, \theta_i)$.
        end for
    end if
end procedure
procedure COMPUTE MEAN PIXEL-WISE RECONSTRUCTION(c_i)
    for each pixel $p = 1$ to P **do**
        $c_p = \frac{1}{K} \sum_{i=1}^{K} c_{i,p}$
    end for
end procedure
procedure COMPUTE PIXEL-WISE UNCERTAINTY(c_i, $c_{\mathbf{p}}$)
    for each pixel $p = 1$ to P **do**
        $\mathrm{Uncertainty}_p = \sqrt{\frac{1}{K} \sum_{i=1}^{K} (c_{i,p} - c_p)^2}$.
    end for
end procedure

Algorithm S2 Computing per-frame metrics from per-pixel uncertainty values

Require: Pixel-wise Uncertainty estimates
Ensure: Inclusion uncertainty (u_{inc}) and relative uncertainty (u_{rel}) metrics, computed per-frame as surrogates for trust attribution

procedure UNCERTAINTY METRICS(Uncertainty)
    Define lesion region R_{inc} based on a segmented mask
    Define background region R_{bkg} as the 5 mm margin surrounding R_{inc}
    Compute mean uncertainty in R_{inc} :

$$u_{\mathrm{inc}} = \frac{1}{|R_{\mathrm{inc}}| \sum_{x \in R_{\mathrm{inc}}} \mathrm{Uncertainty}(x)}$$

    Compute mean uncertainty in R_{bkg} :

$$u_{\mathrm{bkg}} = \frac{1}{|R_{\mathrm{bkg}}| \sum_{x \in R_{\mathrm{bkg}}} \mathrm{Uncertainty}(x)}$$

    Compute relative uncertainty:

$$u_{\mathrm{rel}} = |u_{\mathrm{inc}} - u_{\mathrm{bkg}}|$$

end procedure

Algorithm S3 Frame Selection from multiple acquisitions

Require: N: Total number of acquired frames; $u_{\mathrm{rel},i}$ and $u_{\mathrm{inc},i}$ for each frame i.
Ensure: Selected frames $\mathrm{SI}^{\mathrm{rel}}$ (minimum relative uncertainty) and $\mathrm{SI}^{\mathrm{inc}}$ (minimum inclusion uncertainty)

procedure SELECT FRAME WITH MINIMUM INCLUSION UNCERTAINTY(u_{inc})
    $\mathrm{SI}^{\mathrm{inc}} = \arg \min_{i \in \{1,..,N\}} u_{\mathrm{inc},i}$.
end procedure
procedure SELECT FRAME WITH MINIMUM RELATIVE UNCERTAINTY(u_{rel})
    $\mathrm{SI}^{\mathrm{rel}} = \arg \min_{i \in \{1,..,N\}} u_{\mathrm{rel},i}$.
end procedure

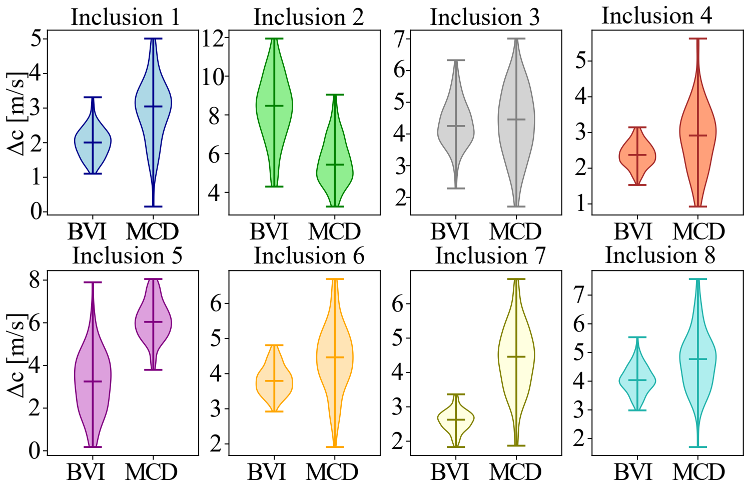

Fig. S3 Δc distributions in 100 samples of BVI and MCD in 8 clinical inclusions to show Gaussianity.

3 Probabilistic Inference Hypothesis in VN for SoS

The studied uncertainty estimation methods rely on multiple samples of a posterior distribution of the model. Fig. S3 shows an example of the Δc distributions acquired from each method on 8 different inclusions of the clinical data. From the Gaussianity of the distribution, we can conclude that the mean and the standard deviation are reasonable estimates for the SoS reconstruction and uncertainty computation.

References

[1] Vishnevskiy, V., Rau, R., Goksel, O.: Deep variational networks with exponential weighting for learning computed tomography. In: Medical Image Computing and Computer Assisted Intervention (MICCAI) Shenzhen, China, pp. 310–318 (2019)

[2] Bernhardt, M., Vishnevskiy, V., Rau, R., Goksel, O.: Training variational networks with multidomain simulations: speed-of-sound image reconstruction. IEEE Trans UFFC **67**(12), 2584–2594 (2020)
